# Radiation shielding for gamma stereotactic radiosurgery units

**DOI:** 10.1120/jacmp.v8i3.2355

**Published:** 2007-08-08

**Authors:** Patrick N. McDermott

**Affiliations:** ^1^ Radiation Oncology Center William Beaumont Hospital Royal Oak Michigan U.S.A.

**Keywords:** radiation shielding, stereotactic radiosurgery

## Abstract

Shielding calculations for gamma stereotactic radiosurgery units are complicated by the fact that the radiation is highly anisotropic. Shielding design for these devices is unique. Although manufacturers will answer questions about the data that they provide for shielding evaluation, they will not perform calculations for customers. More than 237 such units are now installed in centers worldwide. Centers installing a gamma radiosurgery unit find themselves in the position of having to either invent or reinvent a method for performing shielding design.

This paper introduces a rigorous and conservative method for barrier design for gamma stereotactic radiosurgery treatment rooms. This method should be useful to centers planning either to install a new unit or to replace an existing unit. The method described here is consistent with the principles outlined in Report No. 151 from the U.S. National Council on Radiation Protection and Measurements. In as little as 1 hour, a simple electronic spreadsheet can be set up, which will provide radiation levels on planes parallel to the barriers and 0.3 m outside the barriers.

PACS numbers: 87.53.Ly, 87.56By, 87.52Tr

## I. INTRODUCTION

Shielding calculations for the Leksell Gamma Knife (Elekta, Stockholm, Sweden) are complicated by the fact that the associated radiation is highly anisotropic. Shielding evaluation for this device is unique. Although the manufacturer will answer questions about the data that they provide for shielding evaluation, they will not perform calculations for customers. A literature search has not revealed any publications that address shielding design for the Gamma Knife.

As of the end of the year 2005, 237 Gamma Knife centers were in operation worldwide. Centers installing a Gamma Knife find themselves having to either invent or reinvent a method for performing shielding design. Deliberate over‐shielding is commendable; however, it is wasteful to over‐shield because of a lack of proper analytic technique.

This paper introduces a reasonably rigorous, conservative method for barrier design for Gamma Knife model 4C treatment rooms. It should be straightforward to adapt this methodology to other models of gamma stereotactic units. This method should be useful to centers planning either to install a new Gamma Knife unit or to replace an existing unit. The method is consistent with the principles outlined in Report No. 151 from the U.S. National Council on Radiation Protection and Measurements (NCRP).^(1)^ In as little as 1 hour, a simple electronic spreadsheet can be set up, which will provide radiation levels on planes parallel to the barriers and 0.3 m outside the barriers. These values can then be used to decide on the type and thickness of shielding material.

## II. MATERIALS AND METHODS

### A. Treatment unit geometry and source activity

Elekta calls the center of the distribution of sources the unit center. The 201 C60o sources are approximately distributed in a partial spherical shell around this center, which is 1 m above floor level. Consider a coordinate system with origin at the unit center, +z axis vertically upward toward the ceiling, +y axis along the treatment couch (positive toward the foot of the couch), and +x axis perpendicular to the *z* and *y* axes to form a right‐handed coordinate system. Note that the orientation of the Gamma Knife, once established, is not easily changed, given that the mass of the unit is 20,000 kg!

A nominal value of the total initial source activity at installation is 6500 Ci. All shielding calculations in this paper assume an activity of 6500 Ci. The maximum permissible activity is 6600 Ci (Registry of Radioactive Sealed Sources and Devices. Safety evaluation of device no. GA‐269‐D‐102‐S. March 26, 2001). As the sources decay, the radiation levels outside the treatment room will decline.

Barrier design begins by defining, in cooperation with architectural designers, the outer boundaries of the barriers. This first step is simple if the room is rectangular and if the barriers are perpendicular to the coordinate axes as defined earlier.

### B. Air kerma data

The Gamma Knife has three distinct operational states:

1. Shielding doors closed (unit is idle)
2. Shielding doors open while couch is moving in or out
3. Couch in during patient treatment

When the shielding doors are open and the couch is out, radiation levels are at their highest. Elekta provides a matrix (grid) on several horizontal planes listing measured air kerma rates (AKRs) in the vicinity of the unit when the door is open and the couch is out. Here, that quantity is denoted as ${\dot{K}}_{o}$. The units of all air kerma data discussed in this paper are μSv/h.

The AKRs are given on a grid with a spacing of 1 m (except for the plane z=−1). For the couch out, values are given over the range y=−2 m to y=6 m, x=−3 m to x=3 m, and on the planes z=−1.0 m, z=0 m, z=1.0 m, and z=1.5 m. In many cases, one or more boundaries of the treatment room may be located outside this region. In most of the sample plans provided in the Elekta site planning guide,^(2)^ points of interest (0.3 m outside walls) are located outside the boundaries of the air kerma data provided. In addition, Elekta requires a finished ceiling height of no less than 3 m. Furthermore, heating/ventilation/cooling equipment is likely to run above the finished ceiling. The distance from the floor of the treatment room to a height of 0.3 m above the next floor up may easily be 4.5 m (z=3.5 m). Elekta provides no guidance regarding the method to be used to extrapolate AKRs to points outside the supplied grid. This issue is addressed in subsection F.

**Figure 1:**
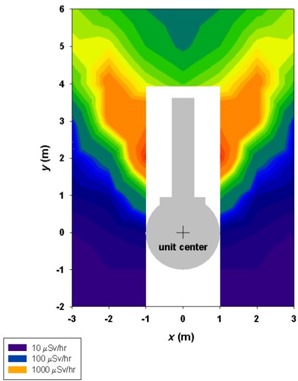
Color‐wash diagram showing radiation levels (in μSv/h) in a horizontal plane (z=0) through the unit center around the unshielded Gamma Knife with the doors open and the couch withdrawn. The source activity is 6500 Ci. No data are available for the white rectangle in the center. Note that the color scale is logarithmic.

**Figure 2:**
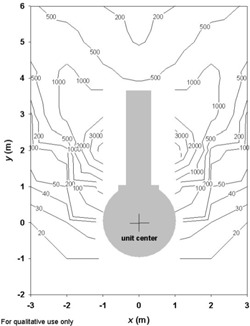
A contour plot showing radiation levels (in μSv/h) in a horizontal plane (z=0) through the unit center around the unshielded Gamma Knife with the doors open and the couch withdrawn. The source activity is 6500 Ci.

**Figure 3:**
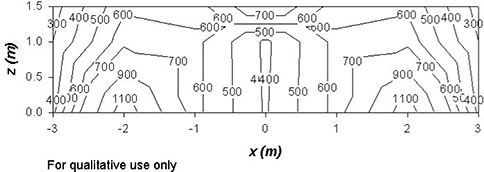
A contour plot showing radiation levels (in μSv/h) in a vertical plane perpendicular to the couch at y=4 around the unshielded Gamma Knife with the doors open and the couch withdrawn. The source activity is 6500 Ci.

**Figure 4:**
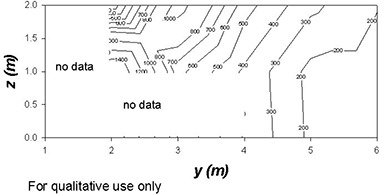
A contour plot showing radiation levels (in μSv/h) in a vertical plane (x=0) along the axis of the couch through the unit center around the unshielded Gamma Knife with the doors open and the couch withdrawn. The source activity is 6500 Ci.

Figs. 1 – 4 show contour plots of the AKRs (couch out) in the planes z=0, x=0, and y=4. These figures are intended for qualitative use only. The plots are based on the grid data provided by Elekta. They give helpful guidance for locating regions where the instantaneous values of ${\dot{K}}_{o}$ are likely to be high.

The AKR data display a certain symmetry about the *y* axis—that is, $\dot{K}(-x,y,z)=\dot{K}(x,y,z)$. On the plane z=0, the contours show high radiation “lobes” (see Figs. 1 and 2) emerging on both sides of the treatment couch at an angle of approximately 25 degrees with respect to the *y* axis. Radiation levels drop off dramatically for angles greater than 55 degrees.

On the elevation view (x=0, Fig. 4), a lobe emerges at an angle of roughly 30 degrees (again with respect to the *y* axis). The spatial distribution of high‐intensity radiation resembles a slightly elliptical hollow cone with y=0 as the axis of the cone. On the z=0 plane, in the high radiation area (0≤θ≤37 degrees), a fitting function has been found that reproduces the data with a root mean square error of about 10%. This function is

(1) $${\dot{K}}_{o}(r,\theta )=\frac{1}{r^{2}}\left[D\;\;e^{-\alpha (\theta^{2}+\theta_{0}^{2})}\cosh(2\alpha \theta \theta_{0})+L\right],$$

where ${\dot{K}}_{o}$ is in units of μSv/h, $D=3.9\times 10^{4},\;\alpha =33,\;\theta_{0}=0.45,\;L=5300$, θ is the angle with respect to the *y* axis (in radians), and *r* is the distance from the unit center in meters.

**Figure 5:**
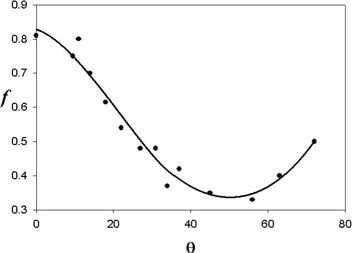
The value of $f={\dot{K}}_{in}/{\dot{K}}_{o}$ as a function of angle with respect to the *y* axis (along the couch). The value of *f* is about 0.5 in the direction of the high‐intensity radiation lobe.

Elekta also provides a matrix of AKR values (for the z=0 plane only) for the situation in which the couch is in the treatment position. Those AKR values are denoted here as ${\dot{K}}_{in}$ Although instantaneous values of ${\dot{K}}_{o}$ are larger than ${\dot{K}}_{\text{in}}$, the values of ${\dot{K}}_{in}$ play a more important role in shielding design than ${\dot{K}}_{o}$ does, because the couch is out only for brief periods of time as compared with the elapsed time during a treatment. As shall be seen later, the ratio of the workloads for these two contributions is roughly a factor of four. It is therefore unfortunate that data for ${\dot{K}}_{in}$ is given only on a single plane. It is expected that there should be a relationship between ${\dot{K}}_{o}$ and ${\dot{K}}_{in}$. Along a radial line from the unit center, ${\dot{K}}_{in}/{\dot{K}}_{o}=f$ is expected, where *f* is like a transmission factor for the couch and the structures attached to the couch. Examination of the ratios ${\dot{K}}_{in}/{\dot{K}}_{o}$ along radial lines indicates that the values of *f* seem to be fairly constant along these lines, based on the data in a single plane provided by Elekta. Where data are unavailable for ${\dot{K}}_{in}$, it is assumed that

(2) $${\dot{K}}_{in}=f(\theta ){\dot{K}}_{o},$$

where θ is the angle between the *y* axis and a radial line to the point of interest (cos θ=y/r, *r* being the distance from the unit center). Fig. 5 shows a graph of *f* (θ) for 0≤θ≤70 degrees. The value of f=0.5 in the direction of maximum radiation intensity (about 25 degrees) and declines to a minimum at about 45 degrees. Values of *f* can be found in Table 1. The values of *f* are averages along ray lines from the unit center. Deviations from the average can be as large as 20%.

**Table 1:** The *f* factor

θ	*f*
0	0.83
5	0.80
10	0.74
15	0.68
20	0.61
30	0.46
40	0.37
45	0.35
50	0.34
55	0.34
60	0.37
70	0.47
75	0.50
90	0.50

The AKR numbers in the matrices provided by Elekta may include leakage, and therefore, in principle, the leakage should be subtracted from the quoted AKRs. The method set out here takes the conservative stance of ignoring this possible “double counting.”

When the shielding doors are closed and the unit is idle, the only radiation is leakage radiation through the shielding. The AKR for this radiation is denoted ${\dot{K}}_{L}$ (in units of μSv/h). Although the level of this radiation is low, it is always present. Elekta reports the amount of this radiation in a document titled *Measured Leakage Radiation That Has Penetrated the External Shielding of the Idle Treatment Unit*.^(2)^ These measurements were made in October 1993 (for a Gamma Knife #30B). The site planning guide also provides a second set of measurements, which are also reported in Attachment 4 of the Registry of Radioactive Sealed Sources and Devices, Safety Evaluation of Device (GA‐269‐D‐102‐S). The measurements in this second set were made in April 1996 for a model B Gamma Knife. Both sets of measurements are provided at 26 points around the shielding “ball” at a distance of 60 cm from the ball's outer surface, which corresponds roughly to a distance of about 100 cm from the nearest source. The measurements have been scaled to a source activity of 6600 Ci. Some significant discrepancies are evident between these two sets of data; at some locations, the discrepancies exceed a factor of two. The more recent data were used here. The values of the AKR are all less than 10 μSv/h with the exception of the measurement point directly outside the shielding doors (on the +y axis), where the value is 28 μSv/h. In this direction, the shielding requirements are overwhelmingly dominated by the operational states 2 and 3 defined at the beginning of this section.

Here, the conservative isotropic value of 10 μSv/h is adopted for ${\dot{K}}_{L}$. The ball is roughly 1.8 m in diameter, and therefore the leakage AKR is assumed to be at a distance of 0.9 m+0.6 m=1.5 m from the unit center. Further, the leakage AKR is assumed to decline as the inverse square of the distance from the unit center, summarized in the equation

(3) $${\dot{K}}_{L}=10{\left(\frac{1.5m}{r}\right)}^{2}\mu \text{Sv}/h\;,$$

where r is the distance in meters from the unit center to the point of interest. As the sources decay, the radiation levels from leakage will decrease.

### C. Barrier thickness

Consider *P* to be the permissible radiation level over a defined period of time, *B* to be the barrier penetration transmission factor, and *K* to be the air kerma at the point of interest. The relationship between these quantities is

(4) $$B=\frac{P}{KT},$$

where *T* is the occupancy factor. The methodology for calculation of *K* at the point of interest is discussed shortly. The necessary thickness *t* for the barrier is

(5) t=−TVL×log(B),

where TVL is the tenth‐value layer thickness for the chosen shielding material.

Examining the data for ${\dot{K}}_{o}$ on the grid, it is apparent that, even for the condition in which the couch is out, the C60o sources are heavily shielded. For the highest AKR [4620 μSv/h at (x, y, z)=(1, 2, 0)], 6500 Ci of exposed C60o at the given distance would lead to an AKR of $1.7\times 10^{7}\;\mu \text{Sv}/h$. Therefore, even under conditions in which the couch is out, and the radiation levels are their highest, this radiation is leakage radiation.

The Elekta‐supplied information indicates that the radiation in the shaded areas (θ>70 degrees) on the matrix diagrams consists of radiation for which “approximately 50% of the dose rate may be radiation that is scattered once.” This statement is of no practical use, given that no TVL value is given for this radiation.

Drzymala et al. investigated the radiation spectrum of a Gamma Knife model 23004B.^(3)^ These authors found that the spectrum is dominated by unscattered C60o photons in the entire region from 20 degrees to 45 degrees with respect to the *y* axis as defined here, but that, at the foot of the couch (θ<20 degrees), the radiation has an energy less than 600 keV. Pending further information, there appears to be no choice but to make the conservative assumption that, for all three operational states of the unit and for all directions, the radiation energy is primary C60o energy.

For convenience, Table 2 provides TVL data for primary C60o radiation. The TVL ratios are 5.2:1.7:1.0 for concrete, steel, and lead respectively. For some barriers, the most intense radiation is incident at a steep angle. In computing the necessary barrier thicknesses, the method presented here ignores that situation and makes the conservative assumption of perpendicular incidence.

**Table 2:** C60o shielding data^a^

	Concrete	Steel	Lead
HVL (cm)	6.2	2.1	1.2
TVL (cm)	21	7.0	4.0

a Assumes primary radiation, from NCRP 151.^(1)^

HVL=half‐value layer; TVL=tenth‐value layer.

### D. Permissible radiation levels

It is an easy matter to use whatever values of *P* are relevant locally. Report No. 151 from the NCRP recommends shielding for controlled areas to a design goal of P=100 μSv per week.^(1)^ This value is lower by a factor of ten than the U.S. Nuclear Regulatory Commission (NRC) limit for radiation workers of 50 mSv per year.^(4)^ For uncontrolled areas, NCRP Report No. 151 recommends a shielding design goal of 20 μSv per week. That goal is equivalent to the NRC limit of 1 mSv per year for individual members of the public. The NRC also requires that the dose equivalent rate in an uncontrolled area not exceed 20 μSv in any one hour. If the occupancy factor is 1.0 for an uncontrolled area, then the weekly permissible level will be the more limiting of the two factors.

### E. Workload

Following Elekta's recommendation, the couch is assumed to move in and out of treatment position an average of four times for each patient treatment (four “shots”).^(2)^ Each of these movements takes 40 – 60 seconds. Each treatment is assumed to take 30 minutes when the source strength is 6500 Ci. The dose rate at the center of a polystyrene sphere of diameter 16 cm is approximately 3.5 Gy/min for 6500 Ci. Therefore the scenario outlined here corresponds to four shots of roughly 25 Gy each. It is assumed that only 1 patient is treated in any given hour. Therefore, during any given hour, the doors will be open and the couch out (operational status 2) for a maximum period of 4×2=8 minutes. If 4 patients are assumed to be treated each day (a very generous assumption), 5 days per week, then the door is open and the couch is withdrawn for a total of 2.7 h per week. These assumptions are extremely conservative and correspond to 1000 patients treated annually. The Leksell Gamma Knife Society reports 13,918 treatments at 89 reporting centers in North and South America in 2005. This is an average of 156 patients per year. Radiation levels will decline as the sources decay; however, the time to move the couch in and out remains constant, and therefore the contribution to radiation levels from operational status 2 will decline with time.

When the couch is in the treatment position, the radiation levels are intermediate between the case in which the shielding doors are closed and the case in which the shielding doors are open and the couch is out. Based on the assumption that 4 patients are treated each day for 30 minutes each, a workload of 10 hours per week for this operating condition (operational status 3) is implied. As the sources decay, the AKR will decline; however, treatment times will rise proportionately, and therefore this contribution to the weekly dose equivalent for operational status 3 will remain constant over time.

Table 3 summarizes the contributions to the workload.

**Table 3:** Workload summary

Operating condition	Workload (h/week)
Idle (state 1)	40^a^
Couch out (state 2)	2.7
Couch in (state 3)	10

a Assumes a standard 40‐hour work week for exposed personnel.

The weekly dose equivalent rate in units of μSv is given by

(6) $$K=40{\dot{K}}_{L}+2.7{\dot{K}}_{o}+10{\dot{K}}_{in}\;\;\;(\text{weekly}),$$

where all instantaneous AKRs are in units of μSv/h.

The hourly dose equivalent is

(7) $$K-\frac{1}{2}{\dot{K}}_{in}+\frac{8}{60}{\dot{K}}_{o}+{\dot{K}}_{L}\;\;\;(\text{hourly}).$$

For the hourly and weekly permissible air kerma values cited in subsection D, the weekly radiation levels will be the limiting values even for the smallest possible occupancy factor (T=1/40). In other words, for the permissible weekly and hourly levels cited above, the barrier thickness will always be determined by the weekly air kerma.

The workload for acceptance testing and commissioning is uncertain. It does not seem likely to be significantly greater than that assumed for the treatment of 20 patients per week. Monthly quality assurance tests are estimated to entail a workload equivalent of about 1 patient, or about 1/80 of the assumed monthly patient load, and is therefore neglected.

### F. Extrapolation of open‐door AKRs

When a point of interest is inside the boundaries of the matrix grid provided by Elekta, then the AKR for that point should be calculated by interpolation in the grid. Linear interpolation in the grid is probably adequate. When a point of interest lies outside the boundaries of the grid, some method of extrapolation must be used. Simply using grid data on the nearest available plane to represent radiation levels at some distance from this plane may be highly inaccurate. Linear extrapolation using the values from the nearest grid plane may also be highly inaccurate because of the strong angular dependence. Before the suggested method of extrapolation is described, the validity of inverse square behavior for the radial dependence of the AKR is considered.

At a distance that is large compared with the size of a radiation source, the intensity of the radiation is expected to decline with the inverse square of the distance from the source. The C60o sources in a Gamma Knife are spread over a partial spherical shell roughly 1 m in diameter. Scattering in the shielding container may make the effective diameter of the source even larger. To the extent that the source is spherical it may *tend* to act like a point source. Unlike a gravitating spherical mass, a spherical radiation source is not precisely equivalent to a point source. Examination of the ${\dot{K}}_{o}$ values at the most distant pair of points along a radial line provides an assessment of the validity of the inverse square law. The most distant pair of points along a ray line are (x,y,z)=(2,4,0) and (3, 6, 0). The values of ${\dot{K}}_{o}r^{2}$ for these points are $24,200\;\mu \text{Sv}\;m^{2}/h$ and $24,300\;\mu \text{Sv}\;m^{2}/h$ respectively, where *r* is the distance from the unit center. Pairs of points that are closer to the unit center show more deviation from inverse square behavior.

The method used here to extrapolate the open‐door AKR to the plane of interest (assumed to be 0.3 m beyond the barrier) is to assume inverse square behavior along ray lines from the unit center. This assumption may slightly underestimate AKR at points outside the provided grid. Inverse square extrapolations of this kind must be performed along ray lines because of the highly anisotropic distribution of AKR. Along some ray lines, one can “see” much further into the unit, and the radiation levels are therefore much higher. This method provides values of ${\dot{K}}_{o}$ on the plane of interest. Upon extrapolating along ray lines, the grid may become rather sparse. It is to be noted that this method may also be applicable to computed tomography shielding, for which manufacturers supply a similar grid of AKR.

To extrapolate to a plane farther from the unit center, data on the nearest plane is typically used. The ceiling always requires such extrapolation, and therefore provides a relevant example. For a horizontal plane at $z=z^{\prime }$, extrapolation proceeds from the plane $z=z_{0}$ along ray lines from the unit center. Based on simple geometry, the grid values of *x* and *y* on the new plane are

(8) $$x\prime =x_{0}\left(\frac{z\prime }{z_{0}}\right),y\prime =y_{0}\left(\frac{z\prime }{z_{0}}\right),$$

and the AKRs at these points are

(9) $${\dot{K}}_{o}(x\prime ,y\prime ,z\prime )={\dot{K}}_{o}(x_{0},y_{0},z_{0}){\left(\frac{z_{0}}{z\prime }\right)}^{2}\cdot$$

Extrapolation for walls follows a similar line of reasoning. A grid of weekly AKR values on the plane of interest is then calculated using equation 6, and shielding is chosen based on the resulting numbers. This approach assumes that the barriers are planar and that the planes are perpendicular to the coordinate axes. This assumption is always true for the ceiling. If the Gamma Knife unit is rotated with respect to the walls, then analytical geometry can be used to generalize the technique outlined above to calculate the intersection location of ray lines with barrier planes.

An example of barrier design for a ceiling follows. The area above the ceiling is an uncontrolled area with an occupancy factor of 1.00. The plane, which is 0.3 m above floor level for the region above the treatment room, is at $z^{\prime }=3.9$ m. This location is considerably above the highest plane for which grid data is available ($z_{0}=1.5$, which is 2.5 m above the floor).

**Figure 6:**
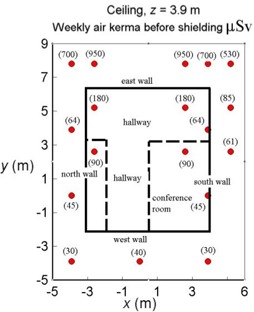
The total weekly air kerma before shielding on a plane $z_{0}=3.9$ m (0.3 m above floor level for the floor above the Gamma Knife). Labels indicate room usage.

Fig. 6 shows values for the total weekly air kerma on the horizontal plane $z^{\prime }=3.9$ m within and around the bounding vertical walls. Data are extrapolated from the planes $z_{0}=1.0$ and $z_{0}=1.5$. The extrapolated values are calculated using equations 9, 8, 6, 3, and 2. The data are somewhat sparse, but they all that are available.

The largest value of $\dot{K}$ within the horizontal boundaries of the walls is 180 μSv. This value is found in the east hallway. The air kerma in this location shows a very high gradient. High gradients should always prompt an examination of air kerma at points just outside boundaries.

At a distance of about 2 m to the east, Fig. 6 shows that the air kerma rises to 950 μSv. A ray line from the unit center to that point actually passes through the east wall before reaching that point, and therefore that value is an overestimate. Nevertheless, to account for uncertainties and in the spirit of “as low as reasonably achievable,” the ceiling is shielded for a weekly air kerma of 950 μSv. If the shielding is adequate for this location, it will be more than adequate for the other locations above the treatment room.

The individual contributions to the total air kerma are $K_{\text{in}}=590\;\mu \text{Sv},\;K_{o}=350\;\mu \text{Sv}$, and $K_{L}=10\;\mu \text{Sv}$. Note that the $K_{\text{in}}$ term dominates. If P=20 μSv per week and an occupancy factor of 1/5 for a corridor are assumed, the necessary barrier transmission is B=0.105. If concrete is used as the shielding material, the required thickness is 21 cm. Alternatively, lead could be used, and in that case, the thickness could be tapered toward the west (see Fig. 6).

A room maze is unnecessary, provided that the door is lateral to and behind the unit center (that is, negative values of *y*). In this case, the leakage radiation is likely to be the dominant contribution to the weekly air kerma. To estimate the leakage contribution at the door more accurately than equation 3 provides, the given value of the leakage radiation at a point that is nearest to a ray line from the unit center to the center of the door can be used. An inverse square correction would be applied to calculate the value at 0.3 m outside the door. Report No. 151 from the NCRP suggests an occupancy factor of 1/8 for the area just outside a treatment room door (see the report's Appendix B).^(1)^

## III. CONCLUSIONS

In as little as 1 hour, an electronic spreadsheet can be set up that can be used to design barrier shielding for the Gamma Knife. The steps to be followed in shielding design are these:

1. In cooperation with the architect, determine the location of the outer boundaries (generally planar) of the treatment room with respect to the location of the unit center and the orientation of the Gamma Knife. The plane of interest is 0.3 m beyond the outside of the barrier.
2. Calculate the instantaneous AKRs (${\dot{K}}_{L},\;{\dot{K}}_{o}$ and ${\dot{K}}_{in}$) over the plane of interest for the three operational modes of the Gamma Knife (shielding doors closed, unit idle; shielding door open, couch out; couch in during patient treatment). Include points just outside the bounding perimeter of the plane of interest to allow for the possibility of high gradients near the bounding edges of the plane. For the leakage contribution, use equation 3. If the plane of interest resides within the grid supplied by Elekta, use linear interpolation to compute ${\dot{K}}_{o}$ and ${\dot{K}}_{in}$. Otherwise, extrapolate along ray lines from the unit center using an inverse square dependence for ${\dot{K}}_{o}$. For an example of the necessary extrapolation, see equations 8 and 9. Calculate ${\dot{K}}_{in}$ from equation 2 and the data in Table 1. The instantaneous AKR will provide guidance for, and a comparison between, survey meter measurements after the facility is built.
3. Use equation 6 to compute the total weekly air kerma over the plane of interest. Fig. 6 shows the result of such a calculation. Apply equations 4 and 5 to determine the necessary barrier thicknesses.

In the future, it would be helpful to have more values of ${\dot{K}}_{in}$, because this term generally represents the dominant contribution to weekly air kerma. It would also be useful to know the TVL for the predominantly scattered radiation lateral to and behind the unit center. This knowledge could lead to a considerable reduction in the necessary barrier thickness.

## ACKNOWLEDGMENTS

The author thanks Robert Drzymala for discussing his paper on the spectrum of the emitted radiation and Di Yan for reviewing this paper. The author also sincerely thanks two anonymous referees who provided useful comments.
